# Study of the Corrosion Behavior of Stainless Steel in Food Industry

**DOI:** 10.3390/ma17071617

**Published:** 2024-04-01

**Authors:** Stefano Rossi, Sergio Maria Leso, Massimo Calovi

**Affiliations:** Department of Industrial Engineering, University of Trento, Via Sommarive 9, 38123 Trento, Italy; sergio.leso64@gmail.com (S.M.L.); massimo.calovi@unitn.it (M.C.)

**Keywords:** stainless steel, biocide, food plant, corrosion, chlorides, bromides

## Abstract

AISI 304L stainless steel is widely used in the processing equipment and food and beverage handling industries due to its corrosion resistance, hygienic properties, and cost-effectiveness. However, it is prone to pitting and crevice corrosion phenomena, the development of which can be influenced by factors such as chloride concentration, temperature, humidity, and bacterial presence. Surface treatments, including roughness levels and residual tensile stress, can significantly affect the corrosion behavior and resistance of the material. This study aims to evaluate the impact of three different surface treatments on the durability of AISI 304L steel. The correlation between surface roughness resulting from pre-treatment and pitting potential values will be examined. Additionally, the influence of different concentrations of biocide additives on surface durability will be assessed to determine the maximum effective concentration for preventing pitting phenomena. Passivation processes will also be evaluated as a potential solution for improving the pitting potential and overall durability of the components. By optimizing surface treatments and biocide concentrations, improved corrosion resistance and durability can be achieved, ensuring the long-term performance and reliability of AISI 304L steel components in critical applications such as food processing and beverage handling.

## 1. Introduction

AISI 304L, which is widely utilized in the processing equipment and food and beverage handling industries [1], is highly favored due to its exceptional resistance to corrosion, which is attributed to the presence of a protective layer comprising chromium oxides [2,3]. Additionally, manufacturers commonly opt for AISI 304L because it meets stringent hygienic standards by facilitating easy cleaning and ensuring that it imparts no taste or color to the processed product [4]. This steel is commonly referred to as “18/8” due to its typical composition of 18 wt.% chromium and 8 wt.% nickel, exhibiting an austenitic microstructure and a face-centered cubic structure. Moreover, it finds applications in carpentry due to its malleability and cost-effectiveness.

The 304L stainless steel grade demonstrates resistance against general or atmospheric corrosion.

It has a tendency to become passive in less harsh environments due to the inclusion of chromium. Moreover, carbon levels are kept minimal to prevent issues during processing, like welding, without the possibility of sensitization and the creation of chromium carbide precipitates. However, it is prone to pitting and crevice corrosion. The susceptibility to these types of corrosion is closely linked to factors such as the concentration of chlorides [5,6], high temperatures, high relative humidity [7], and bacterial presence [8]. In most cases, corrosion occurs when these parameters are present together. Temperature plays a significant role, as the likelihood of pitting and stress corrosion cracking increases with higher water temperatures [9,10]. In industries like food and beverage, where washing and pasteurization involve high temperatures, this becomes particularly relevant. To prevent pitting corrosion, it is crucial to ensure that the concentration of chlorides in the solution in contact with 304L stainless steel does not exceed 150 mg/L [11]. The corrosion potential (E_corr_) and pitting potential (E_pit_), which indicate the tendency for corrosion and localized corrosion, respectively, show a trend toward materials becoming more resistant to corrosion as the concentration of chloride ions decreases at the same temperature [6]. This confirms the importance of controlling the amount of chlorides present. Yeh et al. [12] discovered that the relative humidity (R.H.) in the environment is a factor that influences the initiation of stress corrosion cracking. Stress corrosion cracking refers to the formation and growth of cracks under the combined influence of a corrosive environment and tensile stress on the material. When the chloride concentration is at a threshold value of 0.1 g/m^2^, the R.H. threshold is between 55% and 70%. However, if the chloride concentration is increased to 1 g/m^2^, the R.H. threshold decreases to 45–55%.

The corrosion behavior and resistance of a material are significantly influenced by the surface treatments it undergoes [13,14]. If the finished product has a higher roughness, it means there are deeper grooves on the surface where dust can accumulate, leading to a higher concentration of ions [12]. According to the 3-A sanitation standards, the average surface roughness should be below 0.8 μm in order to be considered “hygienic” [15]. It is important to note that, when the surface roughness (Ra) is lower than 0.5 μm, the steel surface is considered “clean”, indicating the presence of only a few sites where chloride ions can gather [16]. In addition to roughness, the presence of residual tensile stress on the surface also has a significant impact. Turnbull et al. [17] conducted a study on the corrosion of AISI 304L stainless steel, examining four different surface conditions. The researchers found that surfaces with higher tensile residual stress exhibited a greater tendency for pitting corrosion. The presence of residual stresses can promote stress corrosion cracking and contribute to the premature failure of the component.

Satin finish and sandblasting are commonly used for aesthetic purposes in large machine carpentry, and many components in this field have these types of finishes. Sandblasting is known to enhance fatigue resistance and surface hardness [18], resulting in an opaque aesthetic appearance. However, it is important to note that sandblasting processes can negatively affect the corrosion resistance properties of the material [19,20]. In summary, any mechanical surface treatment renders the material’s surface more reactive compared to its solution-annealed condition, since the process partially removes the passivation layer, and also increases the reactive area of the sample [21].

Preventing the adhesion of bacteria, fungi, and algae is of utmost importance for ensuring food safety [22], maintaining quality, and avoiding corrosion-related issues [23]. Ress et al. conducted a study [24] that demonstrated how bacteria can proliferate and deposit on material surfaces, leading to the formation of differential aeration cells, and resulting in early corrosion caused by microbial deposits. In order to prevent bacterial deposition in water, biocide additives are sometimes used. Another approach is to incorporate copper [25] into the composition of 304L stainless steel, as it can reduce the presence of bacteria, corrosion rates, and the tendency for pitting. Improper use of biocide additives can actually contribute to corrosion issues, primarily because of their composition, which may contain halides. The primary disadvantage of oxidizing biocides is their propensity to corrode metals due to their redox potential [26]. However, in economically disadvantaged countries, oxidizing biocides based on chlorides and bromides are often preferred [26,27]. Some commonly used oxidizing biocides include peracetic acid, sodium hypochlorite, and chlorine dioxide, as well as chlorine and bromine-based compounds.

Hence, the objective of this study is to assess how three distinct surface conditions impact the durability of AISI 304L steel. The investigation examines the correlation between the surface roughness levels resulting from the steel surface treatment and the values of pitting potential. Satin finish and sandblasting were selected as surface conditions because they are widely used in large machine carpentry. To emphasize the significance of surface roughness, these conditions were contrasted with the surface morphology of cold-rolled steel, which is recognized for its considerably smoother surface texture. Additionally, this study examines the influence of various concentrations of two common biocides on surface durability, aiming to identify the maximum effective concentration for preventing pitting phenomena. Lastly, the samples undergo a passivation process, which is evaluated as a potential solution for enhancing the pitting potential and improving the overall durability of the components.

## 2. Materials and Methods

### 2.1. Materials and Samples Preparation

The austenitic stainless steel AISI 304L was purchased from CMS Lavorazioni Meccaniche (Schio, VI, Italy). The chemical composition of the material was 18.2% Cr, 8.1% Ni, 0.026% C, 0.4% Si, 1.28% Mn, and 0.032% P, and Fe Bal, as defined by the supplier. Stainless steel sheets (width 2 mm) possess three different surface finishes: sandblasted, cold-rolled, and satin finish. The sandblasting process was carried out with corundum powder (0.2 mm diameter; 70 mesh) and a pressure of 8 bar.

Before testing, the surfaces were pickled using ECOINOX (pH = 1.7) from Delmet (Milan, Italy), washed with de-mineralized water, and rinsed. The composition of the pickling solution is outlined in Table 1, as defined by the supplier. Pickling was performed by the total immersion of the sample for 10 min in a bath containing three parts of water and one part of the pickling solution.

Two of the most prominent biocides employed in the water treatment sector were used in this study. The composition of these biocides is outlined in Table 2, as defined by the supplier. Biocide A was purchased from Solenis (Wilmington, NC, USA), and consists of bromine chloride, which releases chlorine and bromine into the water to function as biocidal agents. Conversely, Biocide B was purchased from Nalco (Naperville, IL, USA), and contains sodium hypochlorite and sodium bromide, which break down in water and generate chlorine and bromine, serving as biocidal agents.

Finally, the passivation treatment was carried out using a 1% citric acid solution in immersion for 30 min. After this process, the specimens were washed and rinsed.

### 2.2. Characterization

Surface roughness was measured with the Mahr MarSurf PS1 roughness meter (Mahr, Göttingen, Germany), to evaluate the effect of the three different surface treatment processes. Five measurements were carried out on five different samples for each series (25 total measurements per series). One specimen of each surface finish was studied using a Scanning Electron Microscope JEOL IT 300 (JEOL, Tokyo, Japan) using back-scattered electrons (BEDs) and secondary electrons (SEDs) in order to inspect the surface roughness visually.

Polarization curves were obtained using an AUTOLAB PGSTAT302N potentiostat (Metrohm Italia, Origgio, VA, Italy) employing an Ag/AgCl with saturated KCl and platinum as reference- and counter-electrodes, respectively. The scan rate was 0.2 mV/s, with a starting potential of −0.05 V, with respect to the corrosion potential, and an ending potential of +1 V, with respect to the corrosion potential. The potential test was conducted using an Avesta Cell, in order to avoid crevice corrosion, on specimens with dimensions 30 mm × 30 mm and an exposed area of 1 cm^2^ at 60 °C. Different concentrations of Biocide A were tested in contact with the sandblasted specimens to find the maximum active bromine concentration to avoid pitting: 2 mg/L, 4 mg/L, 8 mg/L, and 16 mg/L free bromine concentration equivalent to 100 mg, 200 mg, 400 mg, and 800 mg per liter of product. The same tests were performed using Biocide B with the same active concentrations (2, 4, 8, 16 mg/L) equivalent to 300 mg, 600 mg, 1200 mg, and 2400 mg of product. All the tests were performed in an aerated solution. A total of 12 different samples for each series were characterized with this technique (three for each biocide concentration).

Moreover, samples with dimensions of 30 mm × 30 mm were put inside the climatic testing chamber, maintaining the temperature of the solution at 38 °C for 200 h, employing both 2 mg/L and 16 mg/L active concentrations of the solutions containing Biocide A and Biocide B; these were considered to be not-very aggressive and very-severe concentrations, respectively. The design was selected to mimic the wet and dry fluctuations experienced by external components on the pasteurizer. Three samples of each series were characterized for each biocide concentration.

Finally, the protective effectiveness of a passivation process was examined by comparing the pitting potential values before and after treatment. The surface underwent 10 min of immersion for pickling with ECOINOX (pH 1.7) diluted with water at a ratio of 1:3. Subsequently, a passivation treatment was applied using a 1% citric acid solution for a 30 min immersion. Following this process, the specimens were washed and rinsed. Three different samples for each series underwent passivation and were characterized using this technique.

## 3. Results and Discussion

### 3.1. Surface Treatments Characteristics

**Figure 1:**
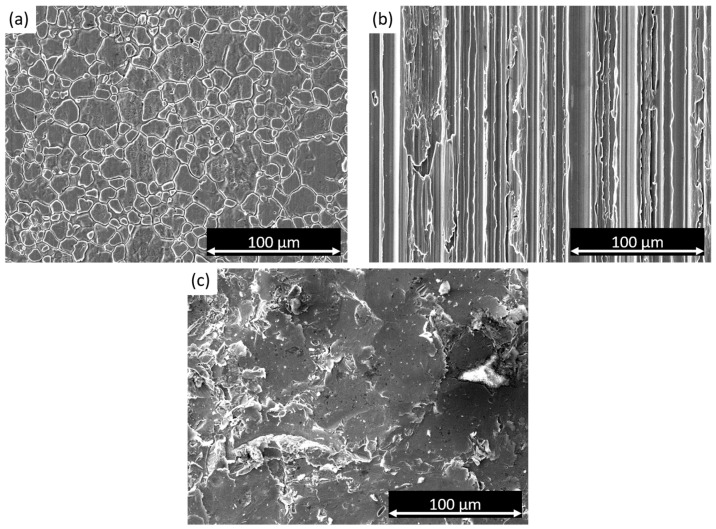
SEM micrographs of (**a**) cold-rolled sample, (**b**) satin finish sample, and (**c**) sandblasted sample.

SEM observations were used to examine the surface morphology of the three samples under investigation, as depicted in Figure 1. Figure 1a exhibits the cold-rolled surface, revealing clearly visible and coarse grains. On the other hand, the satin finish specimen, depicted in Figure 1b, displays a distinct directional pattern resulting from the specific brushing technique used for this surface treatment. It is important to note that satin finish is a superficial treatment that does not induce anisotropic behavior in the sample. Figure 1c presents the sandblasted sample, showing a highly damaged surface indicative of the intensive surface treatment.

Table 3 summarizes the roughness values of the samples, with measurements taken in two perpendicular directions to account for any directional effects caused by certain surface treatments.

The average mean value roughness of the sandblasting treatment is ten times higher than the other treatments, and the Rz value is exceptionally elevated. In the case of the satin finish, the process leads to a horizontal roughness that is twice as high as the vertical roughness. However, the cold-rolled finish stands out as the only treatment with a significantly low roughness value.

### 3.2. Pitting Resistance Evaluation

**Figure 2:**
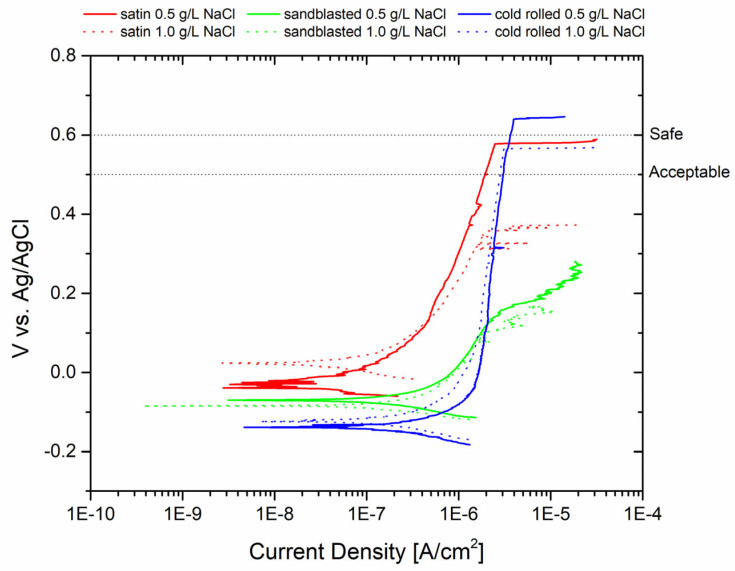
Polarization curves of AISI 304L with different surface treatments in contact with two different aggressive solutions.

Potentiodynamic analyses were carried out in two different test solutions correlating the pitting potential Epit values to the surface roughness of the samples. The results are depicted in Figure 2. The pitting potential values of the samples were as follows: for the sandblasted specimen, it was 0.182 V (0.5 g/L NaCl) and 0.138 V (1 g/L NaCl); for the satin specimen, it was 0.574 V (0.5 g/L NaCl) and 0.343 V (1 g/L NaCl); and for the cold-rolled specimen, it was 0.804 V (0.5 g/L NaCl) and 0.51 V (1 g/L NaCl). In order to assess the suitability of the surface treatments for machinery applications, the figure includes an acceptable and safe pitting potential value. According to the literature [28,29], 0.5 V vs. Ag/AgCl can be considered an acceptable pitting potential value, while 0.6 V vs. Ag/AgCl represents a safe value. As a concentration of 1 g/L of NaCl is considered very high if the plant water is potable (even after biocide addition), it is possible to conclude that the cold-rolled and satin finishes can be considered safe. However, the sandblasted sample is highly susceptible to pitting corrosion.

**Figure 3:**
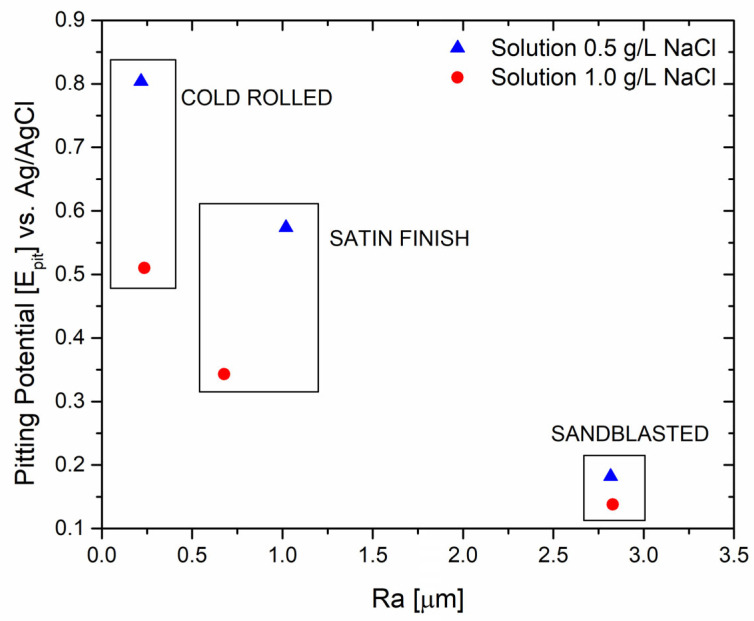
Correlation between average roughness and pitting potential values.

The potentiodynamic test evaluation indicates that sandblasting can be detrimental to corrosion resistance, which can be attributed to the extensively damaged surface of the specimen with a less resistant passive layer. As mentioned earlier, higher roughness reduces corrosion resistance. Roughness measurements were conducted and compared with the pitting potential, as shown in Figure 3, to establish a proper correlation.

**Figure 4:**
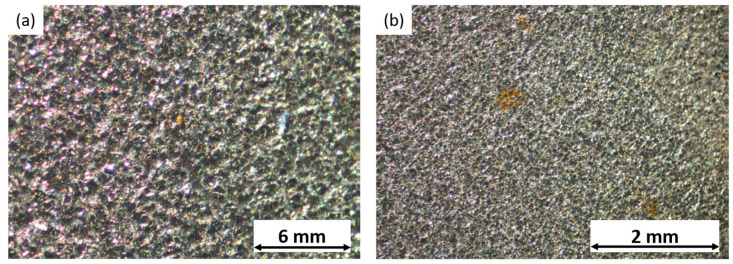
Pitting phenomena observed on sandblasted samples in contact with (**a**) 0.5 g/L NaCl and (**b**) 1.0 g/L NaCl solutions.

Following the testing, the samples were examined under an optical microscope, to observe the pitting corrosion phenomena induced on their surfaces. The resulting pictures confirmed the occurrence of pitting. However, while pitting was present in the cold-rolled and satin finish samples, it was challenging to observe it distinctly using the optical microscope. Thus, Figure 4 represents this phenomenon appreciated in the sandblasted samples.

### 3.3. Biocide Testing

**Figure 5:**
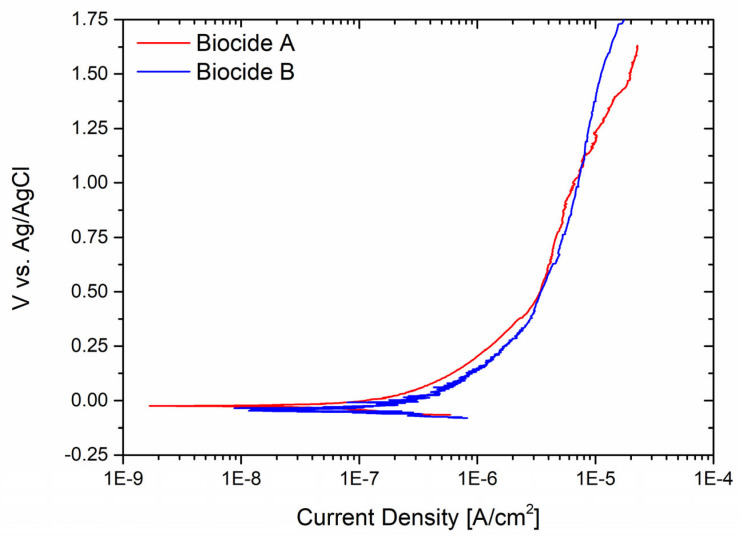
Sandblasted stainless steel corrosion resistance tested using Biocide A and Biocide B with minimum concentration to kill bacteria.

Because the sample that underwent the sandblasting procedure exhibited the highest level of vulnerability to corrosion, its performance was examined when exposed to both oxidant biocides at a concentration of 2 mg/L. This concentration was deemed the minimum requirement for the biocides to be effective, as recommended by the suppliers. Figure 5 shows the potentiodynamic curves of the sample in the two different testing solutions. Both experiments revealed the absence of pitting initiation, suggesting that the two biocides are not corrosive to the metal in such limited concentrations.

**Figure 6:**
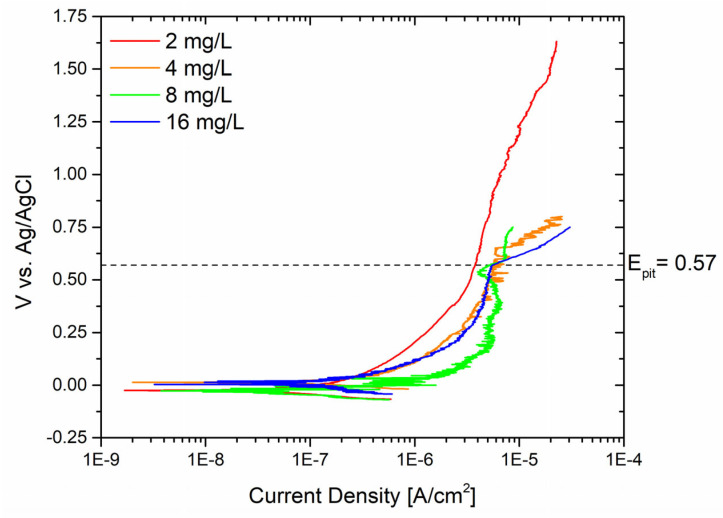
Potentiodynamic curves of sandblasted surfaces with different concentrations of Biocide A.

Thus, Biocide A was examined at various concentrations to investigate the initiation of pitting on the sandblasted sample and to determine the maximum concentration that could effectively prevent pitting phenomena. Indeed, in industrial settings, when it is challenging to precisely control the dosage of biocides, they are often over-applied. This approach ensures that the biocide remains effective throughout the operational lifespan of the plant, reaching its effectiveness threshold. Figure 6 illustrates the results obtained for free bromine concentrations of 2 mg/L, 4 mg/L, 8 mg/L, and 16 mg/L.

Pitting corrosion was not observed at free bromine concentrations of 2 mg/L, as evidenced by the absence of pitting throughout the test until it was stopped at 1.75 V vs. Ag/AgCl. Yet, elevating the concentration of free bromine to 4 mg/L led to the loss of passivity at around 0.62 V vs. Ag/AgCl. A comparable occurrence was noted when employing solutions with concentrations of 8 mg/L and 16 mg/L, resulting in the onset of pitting at 0.52 V and 0.57 V vs. Ag/AgCl, respectively. Hence, it can be affirmed that a free bromine concentration exceeding 2 mg/L proves especially pivotal for the passivity layer of the component. This biocide concentration should thus be regarded as a critical threshold that should not be surpassed to prevent the formation of substantial defects in the metal alloy.

**Figure 7:**
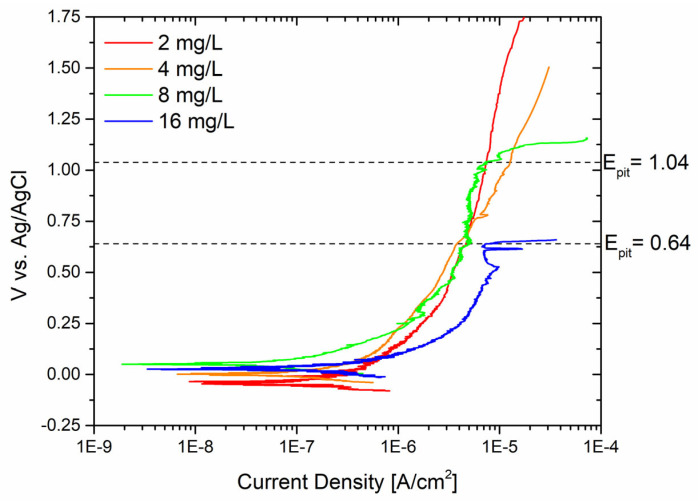
Potentiodynamic curves of sandblasted surfaces with different concentrations of Biocide B.

Similarly, the behavior of the sandblasted sample was evaluated in contact with different amounts of Biocide B, as shown in Figure 7.

Pitting corrosion was observed even at high potentials when the free chlorine concentration exceeded 8 mg/L. The pitting potential Epit was determined to be 1.038 V vs. Ag/AgCl, which is considered a “high” potential value. The threshold for pitting potential in contact with a 16 mg/L free chlorine solution was determined to be 0.64 V vs. Ag/AgCl.

### 3.4. Exposition to Biocide Vapor

The three series of surfaces were subjected to accelerated degradation tests in order to analyze their durability and confirm the output of the previous electrochemical measurements. For this purpose, the atmosphere was obtained by evaporation of both the solution at 2 mg/L and 16 mg/L of Biocide A and Biocide B, respectively.

**Figure 8:**
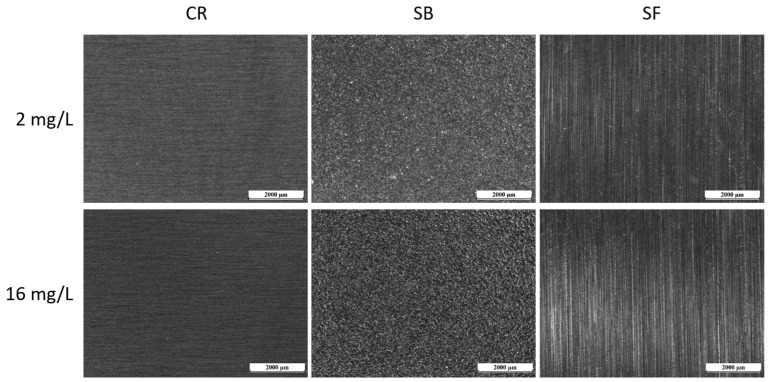
Optical micrographs of the surface of the samples after the test with Biocide A.

Figure 8 illustrates the appearance of samples after conducting a test with Biocide A, where CR stands for ‘cold rolled’, SB ‘sandblasted’ and SF ‘satin finish’. The depicted images represent both tested conditions: the ‘safe’ condition with a free bromine concentration of 2 mg/L and the notably critical condition at 16 mg/L. While the potentiodynamic measurements shown in Figure 6 demonstrated the heightened aggressiveness of Biocide A when applied beyond 2 mg/L of free bromine concentration, the biocide vapor exposure test did not reveal any significant issues for the three surfaces. These surfaces were free from pitting and morphological defects, and, even under particularly high biocide concentrations (16 mg/L), the integrity of the three sample types remained unaffected.

The test results indicate that distinguishing the impact of the various surface processing methods on the steel is challenging, since all three sets of samples remained intact and defect-free. In contrast to immersion in the aggressive biocide solution, exposing the samples to simple steam—mimicking the wet and dry fluctuations typical of external components on the pasteurizer—did not seem to significantly affect the metallic component.

**Figure 9:**
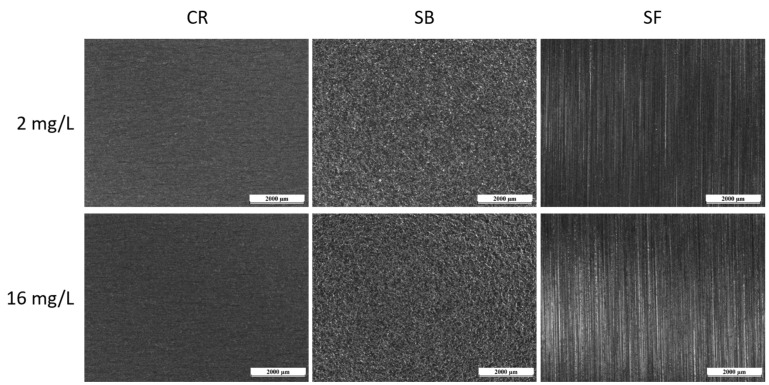
Optical micrographs of the surface of the samples after the test with Biocide B.

Similar findings were observed with Biocide B, as depicted in Figure 9. The images display surfaces that remained intact, without evidence of localized corrosion processes, as anticipated based on the potentiodynamic measurements in Figure 7. Once more, the test conditions proved inadequate for inducing corrosion in the samples, even under Biocide concentrations deemed notably critical. From this standpoint, the surface treatment of the steel, which yields varying morphologies and levels of roughness, does not appear to significantly impact the durability characteristics of the metal component under wet/dry conditions.

### 3.5. Passivation Treatment

Passivation procedures are employed to re-establish the passive layer of stainless steel, particularly when the surface has been damaged and modified by finishing treatments such as shot peening, sandblasting, or other mechanical processing. Prior to passivation, a pickling process is typically performed, either chemically or mechanically. Passivation itself involves the utilization of acids like nitric acid (HNO_3_) or citric acid (C_6_H_8_O_7_). These acids are employed to dissolve the existing passive layer, resulting in the formation of a uniform and protective passive layer enriched with Cr_2_O_3_ after its removal [30]. The pitting potential tends to rise following chemical passivation. In a study conducted by dos Santos et al. [31], the pitting potential of AISI 410 (martensitic stainless steel with 11.50–13.50 wt.% Cr, 0.75 wt.% Ni, 0.08–015 wt.% C) was measured before and after undergoing chemical passivation using nitric acid immersion. The results revealed an increase in the pitting potential, from 155 V vs. Ag/AgCl (for the ground specimen) to 271 V vs. Ag/AgCl, for the chemically passivated specimen. Likewise, recent studies have demonstrated the potential for enhancing the corrosion resistance of various stainless-steel grades used in the food industry. However, it has been noted that 304L steel exhibits a greater vulnerability to localized corrosion compared to other grades [32].

**Figure 10:**
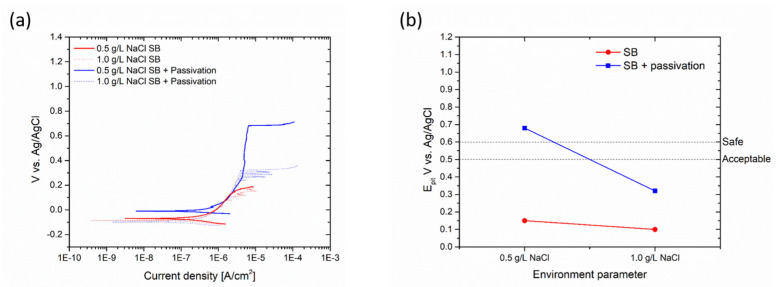
Polarization curves (**a**) and change in pitting potential (**b**) of sandblasted sample due to passivation process.

Thus, polarization curves were obtained for each surface finish of AISI 304L, and subsequent pickling and passivation treatments were carried out to assess any changes in the pitting potential. Figure 10a displays the polarization curves of the sandblasted AISI 304L sample before and after the chemical passivation process, carried out in two solutions with different concentrations of sodium chloride: 0.5 and 1.0 g/L, respectively. Figure 10b illustrates the change in pitting potential due to the passivation chemical treatment. For a 0.5 g/L NaCl solution, the pitting potential can be considered safe for usage. After passivation, the material can be deemed protective when exposed to low to medium concentrations of corrosive elements. Due to the passivation treatment, the pitting potential of sandblasted AISI 304L increased from 0.182 V (0.5 g/L NaCl) to 0.685 V, and from 0.138 V (1 g/L NaCl) to 0.32 V. The passivation treatment elevates the pitting potential, thus providing good protection even to sandblasted specimens when encountering a medium to high concentration of corrosive elements.

**Figure 11:**
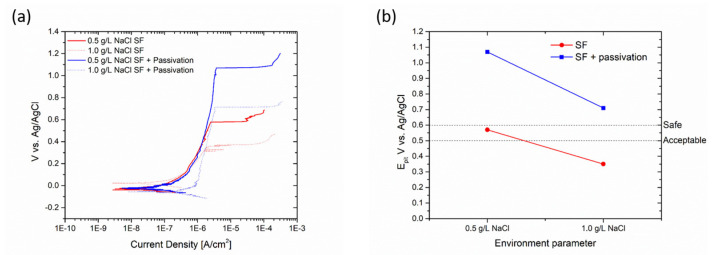
Polarization curves (**a**) and change in pitting potential (**b**) of satin finish sample due to passivation process.

The satin finish sample demonstrated excellent corrosion resistance up to a concentration of 0.5 g/L NaCl, but its performance decreased significantly as the concentration increased. At 1 g/L NaCl, the pitting potential was measured at 0.343 V vs. Ag/AgCl. However, after undergoing passivation treatment, the pitting potential increased to values higher than 0.7 V for both concentrations. The specific changes are depicted in Figure 11.

**Figure 12:**
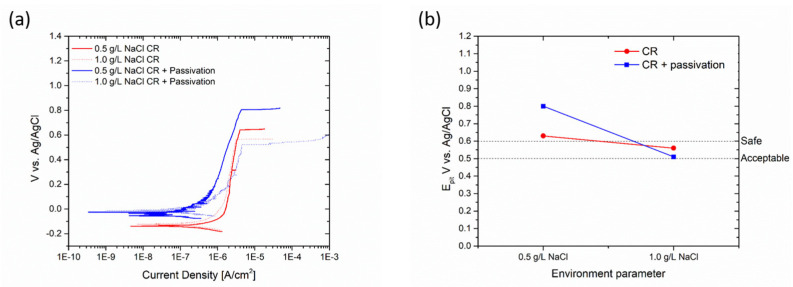
Polarization curves (**a**) and change in pitting potential (**b**) of cold-rolled sample due to passivation process.

The cold-rolled samples consistently exhibited favorable corrosion resistance properties, even under high chloride concentrations. Passivation treatment is not deemed necessary due to the protective nature of the passive layer, as indicated by the pitting potential values of the untreated samples (Figure 12b).

Following the passivation treatment, there was a notable increase in the pitting potential across all surface treatments when immersed in environments containing 0.5 and 1 g/L NaCl. Passivation proved to be particularly effective for the satin and sandblasted surfaces. However, for the cold-rolled surfaces, the pitting potential remained relatively unchanged. This can be attributed to the fact that these kinds of samples undergo minimal manipulation and possess inherent protective properties. The summarized increases in pitting potential are provided in Table 4.

## 4. Conclusions

This work revealed that surface treatments that induce damage to the passive layer of AISI 304L may induce a reduction in the pitting corrosion resistance. For instance, the pitting potential of the sandblasted specimen in a solution with 0.5 g/L of NaCl was equal to 0.182 V vs. Ag/AgCl, compared to 0.804 V vs. Ag/AgCl for the cold-rolled specimen. The average roughness is strictly related to pitting potential, which decreases with an increase in the surface roughness of the material.

In the food industry, tunnel machines are usually subjected to an additive biocidal cycle in order to avoid problems related to foam, bacteria, and scale. Antibacterial additives and external disinfection products can be sources of corrosive elements like bromine and chlorine. The uncontrolled use of these biocides can lead to the corrosion of the components. This study highlights a safety limit found for two different biocides. The AISI 304L sandblasted material can be considered safe if completely immersed in biocide A (bromine-based) with a maximum concentration of 16 mg/L of free bromine, and in biocide B (chlorine-based) with up to 8 mg/L of free chlorine.

The exposure to Biocide vapor was performed to understand if, under service conditions, corrosion occurs in a very short time. The test did not detect any specific defects in the samples and failed to distinguish between the three sets of samples. Nonetheless, the passivation treatment was still investigated to assess potential enhancements in the materials’ pitting resistance, particularly those subjected to mechanical surface treatments. The sandblasted AISI 304L showed an increase in the pitting potential (in 0.5 g/L NaCl) from 0.15 to 0.68 V vs. Ag/AgCl, while the pitting potentials of the satin finish and cold-rolled AISI 304L moved from 0.57 to 1.07 V vs. Ag/AgCl and from 0.63 to 0.80 V vs. Ag/AgCl, respectively. Moreover, this work highlighted that passivation treatment is unnecessary for cold-rolled samples.

## Figures and Tables

**Table 1:** Composition of pickling solution, expressed in weight percentages.

Mixtures	Concentration [wt.%]
Nitric Acid	30 ≤ x < 40
Fluoridric Acid	5 ≤ x < 9.5
Ammonium Bifluoride	2 ≤ x < 5
2-Butoxyethanol	x < 0.1
Acetic acid	x < 0.1

**Table 2:** Biocide A and Biocide B composition expressed in weight percentage.

Biocide A		Biocide B	
Composition	Concentration [wt.%]	Composition	Concentration [wt.%]
Sulphamidic Acid	15–25	Sodium Hypochlorite	7
Bromine Chloride	10–15	Sodium Bromide	10
Sodium Hydroxide	5–10	Sodium Hydroxide	83

**Table 3:** Surface roughness characteristics for each surface treatment.

Surface Finish	Ra [μm]	Rz [μm]
Cold-rolling and pickling (vertical)	0.24 ± 0.03	2.14 ± 0.14
Cold-rolling and pickling (horizontal)	0.27 ± 0.02	2.08 ± 0.15
Sandblasting (vertical)	2.82 ± 0.20	16.95 ± 1.62
Sandblasting (horizontal)	2.83 ± 0.31	17.28 ± 1.86
Satin finish (vertical)	0.68 ± 0.12	4.84 ± 1.32
Satin finish (horizontal)	1.45 ± 0.06	11.18 ± 0.51

**Table 4:** Overview of the pitting potential (V vs. Ag/AgCl) before and after the passivation process.

Surface Treatment	Environment	E_pit_	E_pit_ after Passivation
Sandblasted	0.5 NaCl	0.15	0.68
Sandblasted	1.0 NaCl	0.10	0.32
Satin finish	0.5 NaCl	0.57	1.07
Satin finish	1.0 NaCl	0.35	0.71
Cold-rolled	0.5 NaCl	0.63	0.80
Cold-rolled	1.0 NaCl	0.56	0.51

## Data Availability

The data presented in this study are available on request from the corresponding author. The data are not publicly available due to the absence of an institutional repository.
